# Optical absorption of dilute nitride alloys using self-consistent Green’s function method

**DOI:** 10.1186/1556-276X-9-51

**Published:** 2014-01-29

**Authors:** Masoud Seifikar, Eoin P O’Reilly, Stephen Fahy

**Affiliations:** 1 Tyndall National Institute, Lee Maltings, Dyke Parade, Cork, Ireland; 2 Department of Physics, University College Cork, Cork, Ireland

**Keywords:** Dilute nitride semiconductors, Self-consistent Green’s function, Optical absorption, Band anticrossing model

## Abstract

We have calculated the optical absorption for InGaNAs and GaNSb using the band anticrossing (BAC) model and a self-consistent Green’s function (SCGF) method. In the BAC model, we include the interaction of isolated and pair N levels with the host matrix conduction and valence bands. In the SCGF approach, we include a full distribution of N states, with non-parabolic conduction and light-hole bands, and parabolic heavy-hole and spin-split-off bands. The comparison with experiments shows that the first model accounts for many features of the absorption spectrum in InGaNAs; including the full distribution of N states improves this agreement. Our calculated absorption spectra for GaNSb alloys predict the band edges correctly but show more features than are seen experimentally. This suggests the presence of more disorder in GaNSb alloys in comparison with InGaNAs.

## Background

The substitution of a small fraction *x* of nitrogen atoms, for group V elements in conventional III-V semiconductors such as GaAs and GaSb, strongly perturbs the conduction band (CB) of the host semiconductor. The band structure of dilute nitride alloys has been widely investigated [1]. We have recently developed [2] a SCGF approach to calculate the density of states (DOS) near the conduction band edge (CBE) in these alloys.

One way to test the accuracy of this model is to look at optical absorption spectra for dilute nitride samples, where we expect to see features related to the N states present in the samples. The absorption spectrum arises from transitions between valence and conduction band states. It provides knowledge of the energy gap in semiconductors, and also gives significant information about the band structure of materials. Experimental measurements of absorption spectra can be used to benchmark band structure calculations. In this paper, we investigate two different materials: In_*y*_Ga_1-*y*_N_*x*_As_1-*x*_, for which the band structure has been widely studied and many of the features are well established and GaN _*x*_Sb_1-*x*_, for which much less information has been reported in the literature.

We consider two different models for the band structure of dilute nitride alloys, firstly a five-level band anticrossing (BAC) model, including the host semiconductor CB and valence bands, isolated N and pair N-N states and, secondly, the linear combination of isolated nitrogen states (LCINS) model [3,4], which allows for interaction between N states on nearby sites as well as inhomogeneous broadening and produces a distribution of N state energies. In the LCINS model, the band structure of the alloys is calculated using a SCGF approach [2].

For In_*y*_Ga_1-*y*_N_*x*_As_1-*x*_ alloys, we find that the BAC model reproduces the main features in the absorption spectrum, in agreement with previous work [5,6]. However this model shows some additional features which are related to the N and N-N state energies, reflecting that in the BAC model, we have ignored the actual distribution of localised states. Including the LCINS distribution of N states in In_*y*_Ga_1-*y*_N_*x*_As_1-*x*_ using the SCGF approach [2] removes the additional features found in the BAC calculations and gives absorption spectra that are in very good agreement with experimental data.

We then apply our methods to GaN _*x*_Sb_1-*x*_, where much less information is known theoretically and experimentally. The overall width of the optical spectrum can be well fitted by our models for the absorption spectrum. Both the BAC and LCINS models account for the absorption edge of GaNSb alloys, supporting the presence of a band anti-crossing interaction in these alloys. However, the five-level BAC model gives more features than are seen experimentally in the absorption spectrum. Including a distribution of localised state energies, obtained by modifying those calculated for GaNAs, makes the calculated absorption spectra smoother and gives better agreement with experimental data but still shows some discrepancies around the localised state peak energies. These results suggest the presence of more disorder in GaNSb samples than in InGaNAs. This disorder may be due to sample inhomogeneities or due to an intrinsically broader distribution of N states in GaNSb than in InGaNAs.

The remainder of this paper is organised as follows. In the ‘Methods’ Section we first provide an overview of optical absorption calculation, followed by a description of the band structure models used for dilute nitride alloys. The theoretical results for InGaNAs and GaNSb are presented and compared with experiment in the ‘Results and discussion’ Section. Finally, we summarise our conclusions in the last section.

## Methods

The absorption spectrum *α*(*E*) describes the rate of absorption of photons with energy E=ℏω per unit distance and can be described using a ‘single-electron’ approximation. In this approach, the absorption spectrum for allowed transitions between valence band *v* and conduction band *c* states is given by [7-10]

(1) $$\begin{array}{llll} \alpha_{\text{cv}}(\hbar \omega )\; & = & \;\frac{\pi e^{2}}{n_{r}c\varepsilon_{0}m_{0}^{2}\omega }(f_{v}-f_{c}) & \\ & & \;\underset{k}{\sum }\;M_{b}^{2}\delta \left(E_{\text{ck}}-E_{\text{vk}}-\hbar \omega \right), & \end{array}$$

where ℏω is the photon energy, *e* and *m*_0_ are the electron charge and mass, *c* is the speed of light and *n*_*r*_ is the refractive index. *f* is the Fermi-Dirac distribution function. Here, we assume a filled valence and empty conduction band, so that *f*_*v*_-*f*_*c*_=1. The matrix element in Equation 1 can be written for transitions between valence *p*- and conduction *s*-like zone centre states as [9]

(2) $$M_{b}^{2}=\frac{m_{0}E_{p}}{3},$$

where $E_{p}=(2m_{0}/\hbar^{2})p^{2}$ is the interaction energy, and the momentum interband matrix element, *p*, can be estimated from experiment as

(3) $$p^{2}=\left(1-\frac{m_{e}^{*}}{m_{0}}\right)\frac{\hbar^{2}E_{g}(E_{g}+\Delta_{\text{so}})}{2m_{e}^{*}(E_{g}+\frac{2}{3}\Delta_{\text{so}})},$$

where $m_{e}^{*}$ is the conduction band effective mass, *E*_*g*_ is the band gap (between conduction and valence band) and *Δ*_so_ is the spin-orbit-splitting energy. Therefore, Equation 1 can be written as

(4) $$\alpha_{\text{cv}}(\hbar \omega )=\frac{\pi e^{2}\hbar }{n_{r}c\varepsilon_{0}m_{0}^{2}}\frac{M_{b}^{2}}{\hbar \omega }J_{c,v}(E){\left|\right)}_{E=\hbar \omega },$$

where *E*=*E*_*ck*_-*E*_*vk*_ is the transition energy between conduction (*E*_*ck*_) and valence (*E*_*vk*_) states with wavevector *k*, *J*_*cv*_(*E*) is the joint density of states, and we ignore for now the energy dependence of *M*_*b*_.

The hole-electron interaction can be included assuming Elliot’s theory, which applies to parabolic and nondegenerate bands. According to Elliot’s model [11], the absorption spectrum is modified because of the hole-electron interaction through a multiplicative function *F*_*ex*_ given by [8]

(5) $$F_{\text{ex}}=\frac{2\pi \varsigma }{1-\exp(-2\pi \varsigma )},$$

where

(6) $$\varsigma ={\left(\frac{R_{y}}{\hbar \omega -E_{g}}\right)}^{1/2},$$

and *R*_*y*_ is the exciton Rydberg energy, given by [12,13]

(7) $$R_{y}=\frac{e^{4}}{8h^{2}\varepsilon_{0}^{2}}\left(\frac{\mu }{m_{0}\kappa^{2}}\right)=13.61\left(\frac{\mu }{m_{0}\kappa^{2}}\right)\;\text{eV},$$

where *κ* is the static dielectric constant, *h* is the Planck constant, $\mu =1/(m_{c}^{-1}+m_{v}^{-1})$ is the reduced mass, and $m_{v}={\left(m_{h}^{3/2}+m_{l}^{3/2}\right)}^{2/3}$, where *m*_*h*_ and *m*_*l*_ are the heavy-hole and light-hole effective masses.

Including the hole-electron interaction, the total absorption spectrum *α*_*tot*_(*E*) can then be obtained as

(8) $$\alpha_{\text{tot}}=F_{\text{ex}}\left(\alpha_{\text{lh}}+\alpha_{\text{hh}}+\alpha_{\text{so}}\right),$$

where *α*_*lh*_, *α*_*hh*_ and *α*_*so*_ are the absorption spectra for transitions from the light-hole (LH), heavy-hole (HH) and spin-orbit split-off (SO) bands to the conduction band, respectively.

The effect of the incorporation of N in (In)GaNAs alloys can be described in different ways. We investigate here how the model chosen influences the calculated alloy absorption spectrum. We first present a simple model including isolated and pair N states using the BAC model. This model includes the nonparabolicity of the conduction and light-hole and split-off bands and the interaction between the split-off and conduction bands. In the second model, we then include the full LCINS distribution of localised states using the SCGF model.

### Optical absorption of dilute nitride alloys in five-level BAC model

Here we first consider a simpler model, including isolated and pair N states and their interaction with the host semiconductor conduction, valence and spin-orbit split-off bands. The conventional BAC model treats the host III-V conduction band dispersion as a parabolic band [14]. Test calculations that we have undertaken show that the inclusion of band non-parabolicity can strongly modify the calculated absorption spectra due to the change in the joint density of states caused by the non-parabolicity. In order to treat the host matrix conduction band nonparabolicity and the effect of N on the alloy conduction band dispersion, we construct a 5×5 Hamiltonian. This includes the Kane nonparabolicity of the host matrix conduction band, due to interactions with the light-hole and split-off bands, and treats the effects of N using a three-level BAC model [15], including isolated and pair N states. This Hamiltonian is given as

(9) $$H=\left[\begin{array}{ccccc} E_{c0} & V_{\text{Nc}} & V_{\text{NNc}} & \sqrt{\frac{2}{3}}\text{kp} & \sqrt{\frac{1}{3}}\text{kp}\\ V_{\text{Nc}} & E_{N} & 0 & 0 & 0\\ V_{\text{NNc}} & 0 & E_{\text{NN}} & 0 & 0\\ \sqrt{\frac{2}{3}}\text{kp} & 0 & 0 & E_{v0} & 0\\ \sqrt{\frac{1}{3}}\text{kp} & 0 & 0 & 0 & E_{v0}-\Delta_{\text{so}} \end{array}\right],$$

where *Δ*_*s* *o*_ indicates the spin-orbit-splitting energy, and *E*_*v*0_ is the energy of the valence band maximum. The energy of the isolated N levels (*E*_*N*_), N-N pair states (*E*_*NN*_) and the conduction band edge (*E*_*c*0_) are assumed to vary with composition, *x*, and temperature, *T*, as [15]

(10) $$\begin{array}{ll} E_{N} & =E_{N0}+\gamma_{N}x+a_{N}T,\; \end{array}$$

(11) $$\begin{array}{ll} E_{\text{NN}} & =E_{\text{NN}0}+\gamma_{\text{NN}}x+a_{\text{NN}}T,\; \end{array}$$

and

(12) $$E_{c0}=E_{g}-\gamma_{x}\mathrm{x.}$$

The interaction parameters are assumed to vary as $V_{\text{Nc}}=\beta_{N}\sqrt{x_{N}}$ and $V_{\text{NNc}}=\beta_{\text{NN}}\sqrt{x_{\text{NN}}}$, where the concentrations of single N and N-N pair states *x*_*N*_ and *x*_*NN*_, respectively, are determined from the total N concentration *x* as *x*_*NN*_=6*x*^2^ and *x*_*N*_=*x*-2*x*_*NN*_. The chosen values of the above parameters for (In)GaNAs and GaNSb are given in Tables 1 and 2, respectively [16-18].

**Table 1:** **BAC model parameters for In**_***y***_**Ga**_**1-*y***_**N**_***x***_**As**_**1-*x***_

**Parameter**	**Symbol**	**Values**
N energy	*E*_*N*0_	1.706(1-*y*)+1.44*y*-0.38*y*(1-*y*) (eV)
N-N energy	*E*_*N* *N*0_	1.486(1-*y*)+1.44*y*-0.38*y*(1-*y*) (eV)
d*E*_*N*_/d*T*	*a*_*N*_	-2.5×10^-4^ (eV/K)
d*E*_*NN*_/d*T*	*a*_*NN*_	-2.5×10^-4^ (eV/K)
d*E*_*N*_/d*x*	*γ*_*N*_	-0.22 (eV)
d*E*_*NN*_/d*x*	*γ*_*NN*_	-0.22 (eV)
d*E*_*c*_/d*x*	*γ*_*x*_	-2.1 eV
N interaction	*β*_*N*_	1.97(1-*y*)+2*y*-3.5*y*(1-*y*) (eV)
N-N interaction	*β*_*NN*_	2.69(1-*y*)+2*y*-3.5*y*(1-*y*) (eV)
Energy gap	*E*_*c*0_	*E*_*g*,GaAs_-1.33*y*+0.27*y*^2^ (eV)

**Table 2:** **GaN**_***x***_**Sb**_**1-*x***_** parameters at room temperature**

**Parameter**	**Symbol**	**Values**
Lattice constant [19]	*a*_0_	6.09593 (Å)
Electron effective mass [19]		
Conduction	$m_{c}^{*}$	0.039(*m*_0_)
Light hole	*m*_*l*_	0.0439(*m*_0_)
Heavy hole	*m*_*h*_	0.25(*m*_0_)
Split-off	*m*_*so*_	0.12(*m*_0_)
SO splitting energy	*Δ*_*so*_	0.76 (eV)
Energy gap [20]	*E*_*g*_	0.725 (eV)
Refractive index [19]	*n*_*r*_	3.8
N energy [17]	*E*_*N*0_	0.82-2.3 *x* (eV)
N-N energy [17]	*E*_*N* *N*0_	0.48-2.3 *x* (eV)
N interaction [17]	*β*_*N*_	2.4 (eV)
N-N interaction [17]	*β*_*NN*_	3.39 (eV)

**Figure 1:**
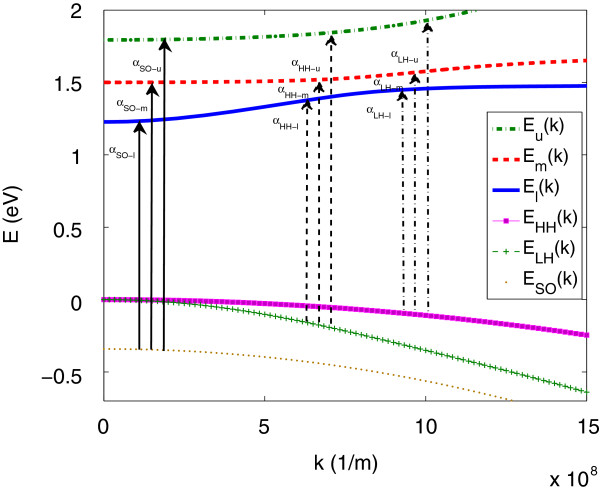
**The band dispersion for In **_**0.04**_**Ga**_**0.96**_**N**_**0.01**_**As**_**0.99**_** in five-level BAC model.** Arrows show the possible optical transition from spin-orbit split-off band (solid arrows), light hole (dashed arrows) and heavy hole (dash-dotted arrows) to conduction sub-bands.

Calculating the eigenvalues of the matrix in Equation 9 gives the dispersion for five bands, namely, the light-hole and split-off valence bands and three conduction bands denoted by *E*_*l*_, *E*_*m*_ and *E*_*u*_ for lower, middle and upper bands, respectively. However, the five-band model of Equation 9 overestimates the LH nonparabolicity and omits the heavy-hole band dispersion. Figure 1 displays the band dispersion for In _0.04_Ga_0.96_N_0.01_As_0.99_ where we have included a parabolic heavy-hole band, $E_{\text{HH}}=E_{v0}-\hbar^{2}k^{2}/(2m_{h})$ and a nonparabolic light-hole band calculated using the six-band Luttinger-Kohn (LK) valence band Hamiltonian [21]. In this model, the band dispersion of the light-hole band is given by

(13) $$E_{\text{LH}}=\frac{E_{l}+E_{\text{so}}}{2}+\sqrt{{\left(\frac{E_{l}-E_{\text{so}}}{2}\right)}^{2}+3{\left(\gamma_{2}\frac{\hbar^{2}k^{2}}{2m_{0}}\right)}^{2}}.$$

where $E_{l}=E_{v0}-\hbar^{2}k^{2}/(2m_{l})$ and $E_{\text{so}}=E_{v0}-\Delta_{\text{so}}-\hbar^{2}k^{2}/(2m_{\text{so}})$ are the parabolic light-hole and spin-orbit bands, respectively, and

(14) $$\gamma_{2}=\frac{m_{0}}{4}\left(\frac{1}{m_{l}}-\frac{1}{m_{h}}\right).$$

The fractional *Γ* character, defined as the contribution of the host matrix CB states to a given level, can be calculated by finding the eigenvectors of Equation 9 and is given by

(15) $$\begin{array}{llll} f_{\Gamma }(E)\; & = & \;\left[1+\frac{V_{\text{Nc}}^{2}}{{\left(E-E_{N}\right)}^{2}}\;+\;\frac{V_{\text{NNc}}^{2}}{{\left(E-E_{\text{NN}}\right)}^{2}}\right) & \\ & & +{\left(\frac{(2/3)k^{2}p^{2}}{{\left(E-E_{v0}\right)}^{2}}+\frac{(1/3)k^{2}p^{2}}{{\left(E-E_{v0}\;+\;\Delta_{\text{so}}\right)}^{2}}\right]}^{-1}. & \end{array}$$

Here, for each conduction sub-band, we use the appropriate energy *E*, as shown in Figure 1 by *E*_*l*_, *E*_*m*_ and *E*_*u*_.

The joint density of states for transitions from the valence band *vi* to the conduction band *cf*, times the host matrix *Γ* character of the conduction state, determines the absorption strength. It is given by

(16) $$J_{\text{cf},\text{vi}}=\frac{k^{2}}{\pi^{2}}\frac{f_{\Gamma }(E_{\text{cf}})}{\left(dE_{f,i}/dk\right)},$$

where $E_{f,i}=\hbar \omega =E_{\text{vi}}-E_{\text{cf}}$ is the energy separation between the CB *cf* and the valence band *vi* states. The absorption spectrum for transitions between bands *vi* and *cf* is given, using Equation 4, by

(17) $$\alpha_{\text{cf},\text{vi}}(\hbar \omega )=\frac{\pi e^{2}\hbar }{n_{r}c\varepsilon_{0}m_{0}^{2}}\frac{M_{b}^{2}}{\hbar \omega }J_{\text{cf},\text{vi}}(\hbar \omega ).$$

Figure 1 represents the band dispersion and all possible optical transitions for In _0.04_Ga_0.96_N_0.01_As_0.99_. The total absorption spectrum is given by the summation of nine individual absorption components, shown in Figure 1, for transitions from VBs *vi* (including LH, HH and SO bands) to CBs *cf* calculated using the 5-level BAC model (*i.e.E*_*l*_, *E*_*m*_ and *E*_*u*_) as

(18) $$\begin{array}{lll} \alpha_{T} & =F_{\text{ex}}\left(\alpha_{l,\text{HH}}+\alpha_{m,\text{HH}}+\alpha_{u,\text{HH}}+\alpha_{l,\text{LH}}+\alpha_{m,\text{LH}}\right)\; & \;\\ & \;+\left(\alpha_{u,\text{LH}}+\alpha_{l,\text{SO}}+\alpha_{m,\text{SO}}+\alpha_{u,\text{SO}}\right),\; \end{array}$$

where *α*_SO-*l*_, *α*_SO-*m*_ and *α*_SO-*u*_ are the absorption spectra from split-off band to lower, middle and upper sub-bands, respectively, and with a similar notation used for transitions from the HH and LH bands.

### Optical absorption of dilute nitride alloys in the LCINS model

In order to include the full distribution of N states, we need to use the Green’s function method in the framework of the LCINS model. In our recent work [2], we showed that the conduction band Green’s function could be written as

(19) $$G_{\text{kk}}(E)={\left\{E-E_{k}-\frac{1}{N_{c}}\underset{j}{\sum }\frac{|V_{j}{|}^{2}}{E-E_{j}-\Delta E_{j}(E)}\right\}}^{-1},$$

where the (complex) energy shift of each localised state *j* is given by

(20) $$\Delta E_{j}(E)=\frac{|V_{j}{|}^{2}}{N_{c}}\underset{k}{\sum }G_{\text{kk}}(E).$$

We can solve Equations 19 and 20 self-consistently by an iterative method [2] to calculate *ΔE*_*j*_(*E*). The density of states per unit volume for the CB, projected onto the host matrix conduction states, is given by

(21) $$D_{\text{cb}}=-\frac{1}{\pi^{2}}\int I\left(G_{\text{kk}}\right)k^{2}d\mathrm{k.}$$

The joint density of states between the CB host matrix components and valence band *vi* can then be obtained using

(22) $$\;J_{c,\text{vi}}(\hbar \omega )=-\frac{1}{\pi^{2}}\overset{k_{\text{max}}}{\underset{0}{\int }}k^{2}I\left[G_{\text{kk}}\left(\hbar \omega +E_{\text{vi}}\right)\right]dk,$$

where *E*_*vi*_ is the energy of the valence band *vi* which, as in the previous section, can be the LH, HH, or split-off (SO) band. We take into account the nonparabolicity of the LH band given by Equation 13, but assume parabolic heavy-hole and split-off bands, as we find that the parabolic split-off band dispersion in the relevant energy range is very close to that obtained when nonparabolicity effects are also included.

Having the joint DOS the optical absorption spectrum can be calculated using

(23) $$\alpha_{\text{tot}}=F_{\text{ex}}\frac{\pi e^{2}\hbar }{n_{r}c\varepsilon_{0}m_{0}^{2}}\frac{M_{b}^{2}}{\hbar \omega }\left(J_{c,\text{LH}}+J_{c,\text{HH}}+J_{c,\text{SO}}\right).$$

The Green’s function given by Equation 19 ignores the nonparabolicity of the host semiconductor CB. In order to consider the Kane non-parabolicity, the Green’s function given by Equation 19 is modified to include the conduction-valence band interaction

(24) $$\;G_{\text{kk}}(E)\;=\;{\left\{E-E_{c}-\frac{p^{2}k^{2}}{E-E_{v}}-\underset{j}{\sum }\frac{|V_{j}{|}^{2}/N_{c}}{E-E_{j}-\Delta E_{j}(E)}\right\}}^{-1}.$$

## Results and discussion

Here, we present the absorption spectra calculated using the five-level BAC and LCINS model and compare them with experiments. Perlin et al. [5,6,22-24] measured the optical absorption spectra for In_0.04_Ga_0.96_N_0.01_As_0.99_ and In_0.08_Ga_0.92_N_0.015_As_0.985_ and compared them with GaAs absorption data. Turcotte et al. [16,25] recently measured the optical absorption spectrum of GaN_*x*_As_1-*x*_ and In_*y*_Ga_1-*y*_N_*x*_As_1-*x*_ for several values of *x* and *y*. Here, we calculate the absorption spectra for In_0.04_Ga_0.96_N_0.01_As_0.99_ and compare them with Skierbiszewski measurements at different temperatures.

### Five-level model for In_*y*_Ga_1-*y*_N_*x*_As_1-*x*_

The interaction between the InGaAs valence and conduction bands and isolated and pair N states in In_*y*_Ga_1-*y*_N_*x*_As_1-*x*_ can be described using Equation 9. The band structure parameters for In _*y*_Ga_1-*y*_As are taken to vary with In composition, *y*, and temperature, *T*, as shown in Tables 1 and 3. Also, the energy and the interaction of isolated and pair N states are taken to vary with In composition as given in Table 1. Figure 1 shows the calculated band structure of In _0.04_Ga_0.96_N_0.01_As_0.99_ where the three conduction sub-bands (*E*_*u*_(*k*), *E*_*m*_(*k*) and *E*_*l*_(*k*)) are determined as the eigenvalues of Equation 9. Also, we consider the lowest eigenvalue of Equation 9 as the split-off band energy (*E*_SO_). The non-parabolic light-hole (*E*_LH_) is given by Equation 13, and the heavy-hole band (*E*_HH_) has been taken to be parabolic.

**Table 3:** Electrical and optical parameters in GaAs

**Parameter**	**Symbol**	**Values**
Lattice constant [19]	*a*_0_	5.65+3.88 ×10^-5^ (*T*_*e*_-300) (Å)
Electron effective mass [13]		300 K/10 K
Conduction	$m_{c}^{*}$	0.063/ 0.067(*m*_0_)
Light hole	*m*_*l*_	0.076/ 0.082(*m*_0_)
Heavy hole	*m*_*h*_	0.50/ 0.51(*m*_0_)
Split-off	*m*_*so*_	0.145/ 0.154(*m*_0_)
Energy gap (*T*=0)	*E*_*g*0_	1.519 eV
Varshni parameters [19]		
$\left(E_{g}=E_{g0}-\frac{\alpha_{T}T_{e}^{2}}{\left(\beta_{T}+T_{e}\right)}\right)$	*α*_*T*_	5.408×10^-4^ (eV/K)
	*β*_*T*_	204
SO splitting energy [19]	*Δ*_*so*_	0.34 eV
Refractive index [13]	*n*_*r*_	3.255(1+4.5×10^-5^*T*_*e*_)
Static dielectric constant [26]	*κ*	12.4(1+1.2×10^-4^*T*_*e*_)

**Figure 2:**
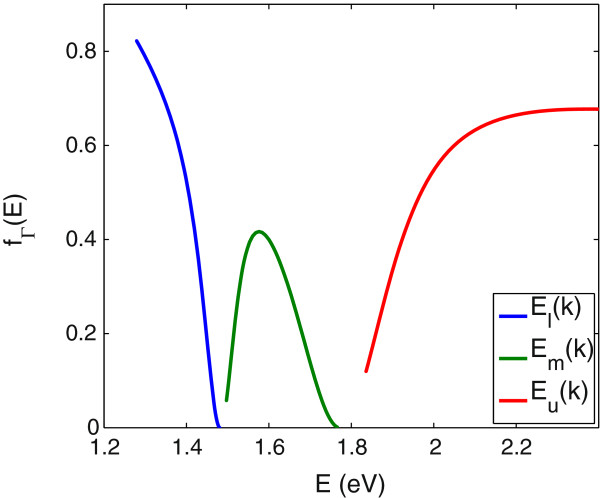
**The fractional*Γ* character for In**_***y***_**Ga**_**1-*y***_**N**_***x***_**As**_**1-*x***_** with*y*=4*%* and*x*=1*%*.** The fractional *Γ* character calculated using the five-level BAC model for conduction sub-bands, at *T*=10 K.

The fractional *Γ* character of the conduction sub-bands is also required in order to calculate the joint density of states between the *Γ*-like conduction band components and the valence bands. Figure 2 shows the calculated *Γ* character of the CB for In _0.04_Ga_0.96_N_0.01_As_0.99_, obtained using Equation 15. It is observed that in the lower sub-band, *f*_*Γ*_ has its maximum value at the CBE and decreases toward zero at the top of the lowest band. It increases again from zero to a maximum value of around 0.4 and goes back to zero in the middle band. Then in the upper band, it increases gradually from its minimum at the bottom of the upper band, approaching an approximately constant value around *E*=2.1 eV.

**Figure 3:**
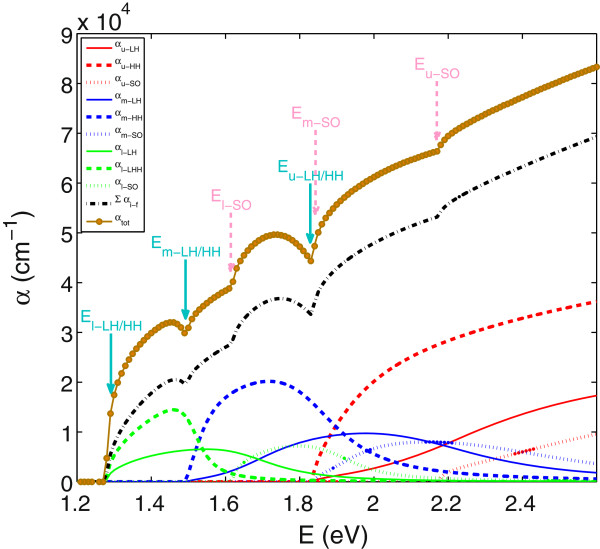
**The absorption spectrum for In**_***y***_**Ga**_**1-*y***_**N**_***x***_**As**_**1-*x***_**, with*y*=4*%* and*x*=1*%* calculated using the five-level model at*T*=10 K.** The contribution of the transitions between three valence bands and three conduction sub-bands are shown. The solid arrows designate the transitions from HH and LH bands to lower (l), middle (m) and upper (u) conduction sub-bands. The dashed arrows indicate the transitions from spin-orbit split-off band to conduction sub-bands.

Figure 3 shows the calculated contributions of the different transitions to the total absorption spectrum. The solid, dashed and dotted lines in this figure represent the contributions due to transitions from the LH, HH and SO bands, respectively, to the conduction sub-bands. The red, blue and green lines indicate transition to the upper, middle and lower conduction sub-bands, respectively. The summation of these nine transitions is shown by the black dash-dotted line in this figure. Multiplying this by *F*_*ex*_ gives the total absorption spectrum shown by the brown circles in this figure.

The usual BAC model predicts a gap in the DOS [2,27] of (In)GaNAs alloys. However, it is clear from Figure 3 that the joint DOS for different transitions overlaps and fills this gap. Therefore, no gap is found in the absorption spectrum in (In)GaNAs alloys when using the BAC model.

**Figure 4:**
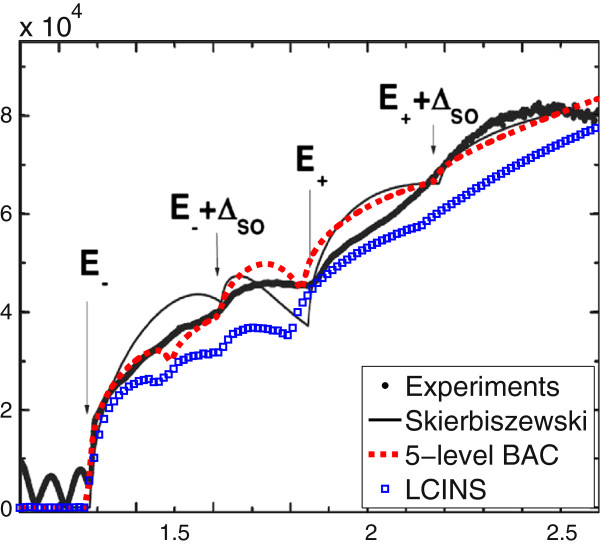
**The absorption spectrum for In**_***y***_**Ga**_**1-*y***_**N**_***x***_**As**_**1-*x***_** with*y*=4*%* and*x*=1*%*.** The red dashed line and blue squares represent the absorption spectrum calculated using the five-level BAC model and the SCGF including the LCINS distribution of localised states (obtained for GaN _0.012_As_0.988_), respectively. The black dots and line show the measured and calculated spectrum by Skierbiszewski [5] at temperature 10 K.

Figure 4 compares the calculated absorption spectrum using the five-level BAC model with that measured and calculated by Skierbiszewski [5]. The black dots in this figure are the experimental results for the absorption spectrum of In _0.04_Ga_0.96_N_0.01_As_0.99_ at *T*=10 K. The solid black line shows the calculated absorption coefficient using the two-level BAC model [6] with constant *V*_*Nc*_=2.7 eV and *E*_*N*_=1.65 eV. This line shows some discrepancies with the experimental data, especially around the transition to the upper sub-band of the BAC model. The arrows in this figure indicate the different transitions from the split-off, heavy- and light-hole band edges to the lower and upper sub-band edges, in the BAC model. The dashed red line in this figure displays the absorption spectrum calculated using the five-level BAC model, which shows much better agreement with the experimental measurements. This model still shows some steps corresponding to the transitions from the HH and LH band to the lower, middle and upper conduction sub-bands (see Figure 3), whereas the experiment shows a much smoother absorption spectrum and has only one pronounced step around *E*=1.85 eV. This is due to the fact that in the five-band model, we have considered isolated and pair N states. Considering the full distribution of N states makes the calculated absorption spectrum smoother, in better agreement with the experimental data.

### LCINS approach for In_*y*_Ga_1-*y*_N_*x*_As_1-*x*_

**Figure 5:**
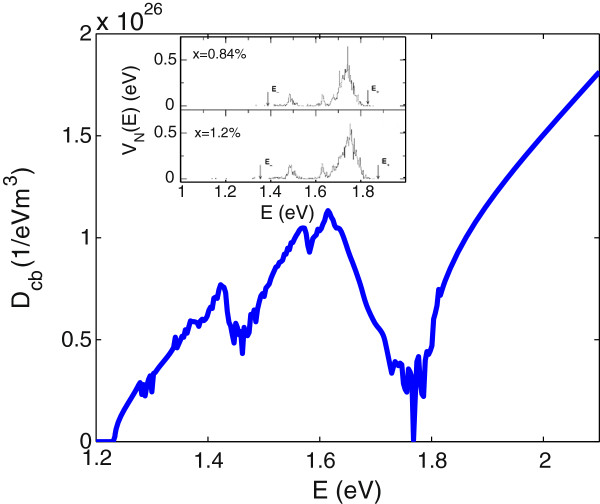
**The density of state for In**_***y***_**Ga**_**1-*y***_**N**_***x***_**As**_**1-*x***_**, with*y*=4*%* and*x*=1*%*.** Density of states calculated using the SCGF approach and LCINS distribution of N states at room temperature. Inset shows calculated distribution of N cluster state energies at low temperature, weighted by their interactions with the conduction band edge state for GaN _*x*_As_1-*x*_ with *x*=0.84*%* and *x*=1.2*%*.

In order to calculate the absorption spectrum using the LCINS model, we first calculate the Green’s function for the CB, given by Equation 24. The inset in Figure 5 shows histograms of the distribution of localised states for GaN _*x*_As_1-*x*_ with *x*=0.84*%* and *x*=1.2*%*, calculated using the LCINS approach [3,4]. This figure shows that the LCINS distributions for *x*=0.84*%* and *x*=1.2*%* are very similar. This implies that the LCINS distribution for GaN _*x*_As_1-*x*_ with *x*=1.0*%* can be approximated by the one for *x*=1.2*%*. However, the calculated CBE at *x*=1.2*%* (indicated by *E*_-_) is about 30 meV lower than the CBE for *x*=0.84*%*. In the five-band BAC model, we assumed that the energy gap in In _*y*_Ga_1-*y*_As is given by *E*_*g*,*G* *a* *A* *s*_-1.33*y*+0.27*y*^2^. Also, the BAC model parameters in Table 1 suggest that including 4% In in In _0.04_Ga_0.96_N_0.01_As_0.99_ reduces the interaction *V*_*N*_(*E*) by about 5%, which we ignore in the LCINS model. Because we do not have the LCINS distribution for In _0.04_Ga_0.96_N_0.01_As_0.99_, we approximate it here by the LCINS distribution calculated for GaN _0.012_As_0.988_.

We then solve Equations 20 and 24 self-consistently [2]. Figure 5 shows the CB DOS calculated by Equation 21 for In _0.04_Ga_0.96_N_0.01_As_0.99_ alloy. We observe that use of the LCINS distribution of states inhibits the gap predicted by the BAC model in the DOS of (In)GaNAs alloys.

The blue squares in Figure 4 show the calculated absorption spectrum at *T*=10 K including the full LCINS distribution of N states, which is compared with the absorption spectrum measured by Skierbiszewski [5]. Clearly, the sharp steps that we saw in the five-level BAC model disappear due to the inclusion of the distribution of localised states. This gives a better overall agreement with the experimental data. The remaining discrepancies between the calculated and experimental data may be partly due to the fact that we have approximated the N distribution by the one that was previously calculated for GaN _0.012_As_0.988_.

**Figure 6:**
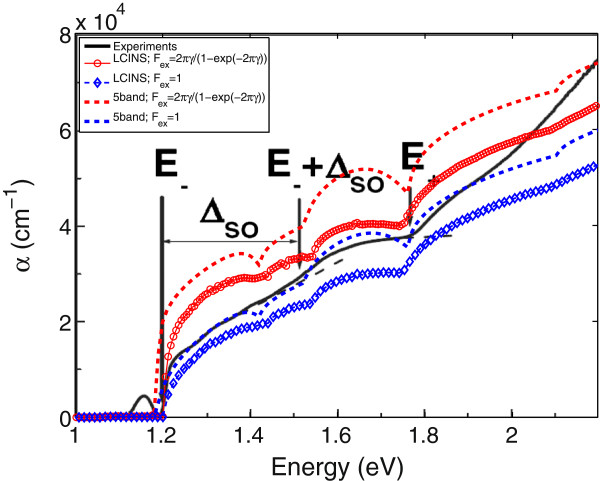
**Room temperature absorption spectrum for In **_**0.04**_**Ga**_**0.96**_**N**_**0.01**_**As**_**0.99**_** calculated using LCINS and five-level models.** The absorption spectrum calculated using the LCINS (red circles and blue diamonds) and five-level BAC (dashed lines) models. The red and blue curves display the results with and without including the electron-hole interaction. The solid black line shows the experimental data.

The room temperature absorption coefficient calculated from the SCGF method including the full LCINS distribution of N states is shown in Figure 6. The solid black line in this figure displays the absorption spectrum measured by Skierbiszewski [5] at *T*=300 K. The red circles in this figure show the absorption spectrum calculated in the LCINS model, where *F*_*ex*_ is calculated using Equation 5. The blue diamonds here indicate the result when we consider *F*_*ex*_=1. The dashed blue and red lines in this figure show the optical absorption calculated using the five-level BAC model, assuming *F*_*ex*_=1 and given by Equation 5, respectively. Here, we again observe that the results calculated using the LCINS model have lower values in comparison with those calculated by the five-level BAC model. This is because of the differences in the band non-parabolicity that we have considered for the valence bands, in the LCINS and five-level LCINS models. Figures 4 and 6 suggest that *F*_*ex*_ might have a stronger temperature dependence than what we have considered in our calculation. The temperature dependence is considered only in the static dielectric constant as shown in Table 3 in calculating exciton Rydberg energy in Equation 6.

### The absorption spectrum for GaN _*x*_Sb_1-*x*_

We can apply our method to calculate the band structure and absorption spectrum of other dilute nitride alloys. Here, we extend our calculations to investigate the absorption spectrum of GaNSb. The room temperature band gap of GaSb is about 725 meV, around half that of GaAs. Lindsay et al. [17,28] have reported that N-related defect levels lie close to the CBE in GaNSb and therefore strongly perturb the lowest conduction states in this alloy. The band gap and optical properties in GaN _*x*_Sb_1-*x*_ have been shown to be strongly affected and highly sensitive to the distribution of the nitrogen atoms. Lindsay et al. [28] found that there is a wide distribution of N levels lying close to and below the CBE. The higher-lying N states push the CBE down in energy, as in GaAs, but the large number of lower energy N states are calculated to mix in strongly with the conduction band edge states, severely disrupting the band edge dispersion in GaNSb.

Here, we first investigate the band structure and optical absorption spectra of GaN _*x*_Sb_1-*x*_ in the five-level BAC model and compare the results with the absorption spectra measured by Veal et al. [20] and Jefferson et al. [29]. We then apply the SCGF method to GaN _*x*_Sb_1-*x*_ in the Section ‘LCINS model for GaN _*x*_Sb_1-*x*_’. As the LCINS distributions have not yet been calculated for these alloys, we modify those calculated for GaNAs alloys and use them in our calculations.

### Five-level BAC model for GaN _*x*_Sb_1-*x*_

When a single Sb atom is replaced by N in GaSb, the N atom introduces a localised state with energy *E*_*N*_. However, a GaNSb alloy can also contain clusters of N atoms, such as N-N nearest neighbour pairs as well as larger clusters that introduce states in the band gap of GaSb. Table 2 contains the band parameters that we use for GaN _*x*_Sb_1-*x*_, including the isolated N state energies, *E*_*N*_ and N pair state energies, *E*_*NN*_ relative to the valence band maximum energy and the BAC interaction parameters *β*_*N*_ and *β*_*NN*_. As shown in this table, isolated N states are calculated to be less than 0.1 eV above the conduction band minimum, while the N pair states have energies that lie in the GaSb band gap. The calculated energy gap of GaN _*x*_Sb_1-*x*_ depends strongly on the assumed N distribution, reflecting that N cluster states introduce a series of defect levels close to the CBE in this alloy. In addition, the interaction parameters (*β*_*N*_ and *β*_*NN*_) in GaN _*x*_Sb_1-*x*_ are calculated to be about 20% larger than for GaNAs alloys.

**Figure 7:**
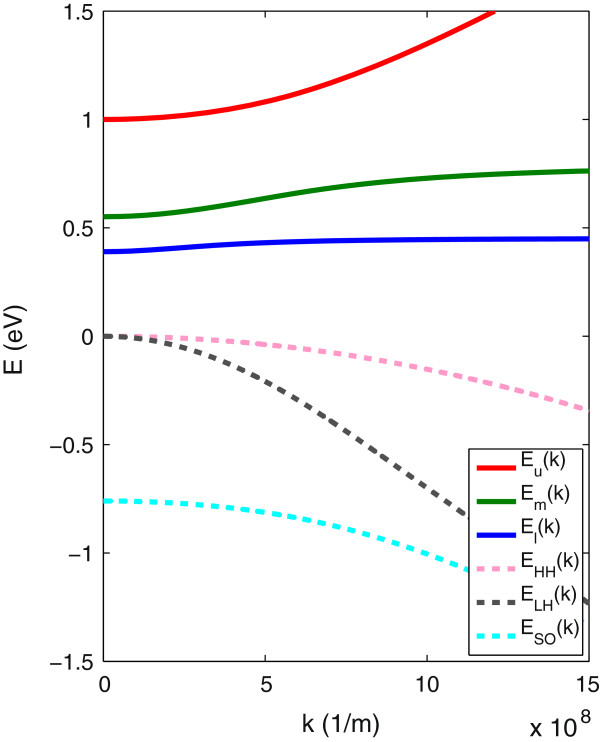
**The band dispersion of GaN **_**0.012**_**Sb**_**0.988**_** calculated by the five-level BAC model.** The solid lines display the conduction bands including upper (*E*_*u*_), middle (*E*_*m*_) and lower (*E*_*l*_) sub-bands. The dashed lines show the spin-orbit split-off (*E*_SO_), light-hole (*E*_LH_) and heavy-hole (*E*_HH_) bands.

Figure 7 shows the conduction and valence band dispersion, calculated using the five-level BAC model given by the Hamiltonian of Equation 9. The solid lines in this figure show the conduction sub-bands. Here, we include the isolated and pair N states and their interaction with the GaSb conduction and valence bands, as explained in the previous section. Since *E*_*N*_ is very close to the GaSb CBE and *E*_*NN*_ just below it, we observe that the lower sub-band (*E*_*l*_) is almost flat and located within the GaSb band gap. The band edge minimum for this band is 0.39 eV and its maximum energy is 0.45 eV. This implies that substitution of only *x*=1.2*%* N by Sb in GaSb reduced the energy gap rapidly from 725 to 390 meV. This value for the band gap of GaN _*x*_Sb_1-*x*_ with *x*=1.2*%* is very close to that which was previously measured [20] and calculated using *k.p*[28] and *ab initio* pseudopotential [30] calculations.

The middle sub-band (*E*_*m*_) lies between 0.55 and 0.78 eV, and the upper sub-band (*E*_*u*_) minimum is close to 1.0 eV. The blue dashed line in Figure 7 shows the non-parabolic spin-orbit split-off band (*E*_SO_), calculated by the lowest eigenvalue of Equation 9. Also, the non-parabolicity of the light-hole band (*E*_LH_) has been taken into account using Equation 13, while we assumed that the heavy-hole (*E*_HH_) band has a parabolic dispersion.

**Figure 8:**
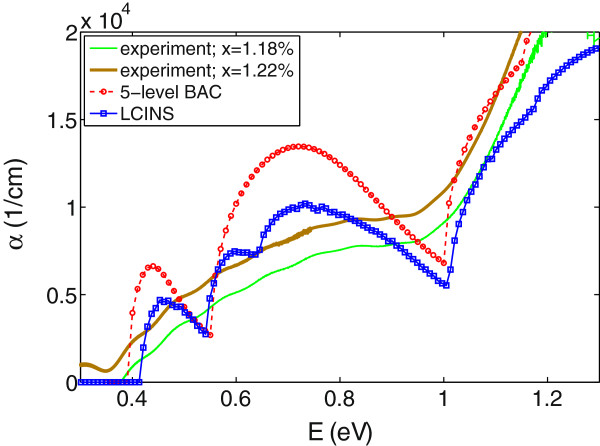
**The absorption spectrum of GaN **_**0.012**_**Sb**_**0.988**_**.** The absorption spectrum for Gan _*x*_*Sb*_1-*x*_ with *x*=1.2*%*, calculated using the SCGF model and including the distribution of localised states (blue diamonds), in comparison with experimental data measured for GaN _*x*_Sb_1-*x*_ with *x*=1.18*%* and *x*=1.22*%* (solid lines). The results calculated by the five-level BAC model are displayed by the red line with circles.

Given the band dispersion, we can calculate the optical absorption as described earlier for InGaNAs. The red dashed line (with circles) in Figure 8 shows the absorption spectrum of GaN _0.012_Sb_0.988_ calculated using the five-level BAC model. Green and brown solid lines in this figure show the absorption spectra measured by Mudd et al. [31] for GaN _*x*_Sb_1-*x*_ with *x*=1.18*%* and *x*=1.22*%*. Our calculated absorption edge is in good agreement with these experiments. However, there are two sharp steps in the calculated spectra corresponding to transitions from the light- and heavy-hole bands to the middle and upper sub-bands. We observe that the experimental absorption spectrum, *α*(*E*), increases from zero at the CBE, to about 3×10^3^ cm ^-1^ at energy *E*=0.55 eV, that is the calculated band edge of the middle sub-band. After this point the slope of the spectra decreases up to *E*=1 eV, the minimum of the upper conduction sub-band. Then, due to the transition from valence bands to the upper conduction sub-band, the magnitude of the calculated absorption spectra increases rapidly. The spin-orbit splitting energy, *Δ*_so_, is 0.76 eV. Therefore, transitions from the split-off band commence at 1.15 eV, where we see a small increase in the calculated absorption spectrum due to transitions from the spin-orbit split off band to the lowest conduction sub-band *E*_l_. The calculated optical absorption using a 5×5 k.p Hamiltonian, accounts well for the absorption edge. Wang et al. [32] have also measured the absorption edge of GaN _*x*_Sb_1-*x*_ with *x*=0.3*%*, *x*=0.7*%* and *x*=1.4*%*, with the measured band edge energies in very good agreement with those calculated by the five-level model of this paper.

We note however that there are two sharp features in the calculated results that are not observed in experiments. This could be due to the fact that we included only isolated and pair N states in this model and ignored the distribution of N states and their inhomogeneous broadening. The results for the calculated absorption spectrum using a five-level BAC model suggest that we need to include the full distribution of N states in optical absorption calculations.

### LCINS model for GaN _*x*_Sb_1-*x*_

It has been shown that the calculated electronic structure of GaN _*x*_Sb_1-*x*_ strongly depends on the assumed distribution of N atoms [28]. Therefore, in order to calculate an accurate band dispersion for this alloy, we need to have the distribution of localised states. Unfortunately, such a distribution has not been calculated for GaN _*x*_Sb_1-*x*_. However, we expect that the distribution of N states in GaN _*x*_Sb_1-*x*_ should have a similar general form to the LCINS distribution that Lindsay et al. [3] have calculated for GaN _*x*_As_1-*x*_ and for GaN _*x*_*P*_1-*x*_ alloys [17]. Therefore, here, we consider the LCINS distribution of N states in GaN _*x*_As_1-*x*_ and, with some small modifications, use that for GaN _*x*_Sb_1-*x*_ alloys.

**Figure 9:**
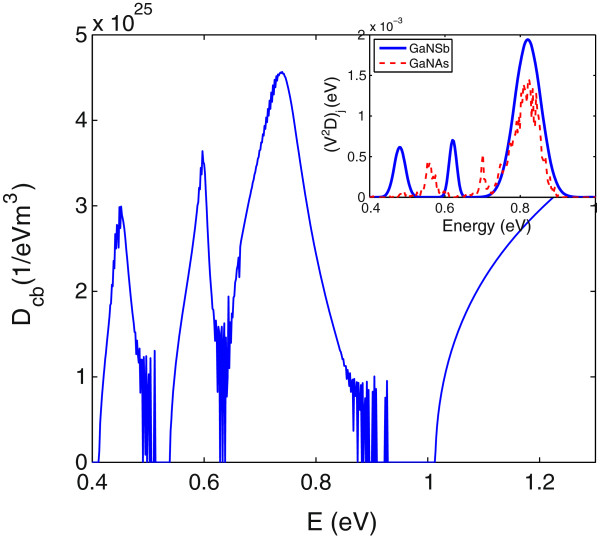
**The DOS of GaN **_**0.012**_**Sb**_**0.988**_**, calculated using the SCGF method and including the LCINS distribution given in the inset.** Inset displays the distribution of N states assumed for GaN _0.012_Sb_0.988_ (blue line), in comparison with the LCINS distribution of GaN _0.012_As_0.988_, shifted down in energy by 888 meV (dashed red line). The zero of energy is taken to be at the top of the GaSb valence band.

In the previous section, we have seen that the calculated energy of an isolated N state *E*_*N*_ is at about 0.82 eV. So, we first need to shift the LCINS distribution of GaN _0.012_As_0.988_ to locate the highest peak at this energy. The dashed red line in the inset of Figure 9 displays the LCINS distribution of N states, weighted by $V_{j}^{2}/\sqrt{N_{c}}$, calculated for GaN _*x*_As_1-*x*_ with *x*=1.2*%*, and shifted down in energy by 888 meV. This distribution can be approximated by three Gaussian distributions, each corresponding to different N environments. It is observed in this figure that if we align the main peak at *E*=0.82 eV, the lowest peak corresponding to pair N-N states is located at 0.55 eV, which is higher than the values that we considered for *E*_*NN*_ in the BAC model. Therefore, we shift the Gaussian distributions corresponding to pairs and larger clusters of N states down by a further 70 meV. Moreover, the BAC model parameters in Table 2 suggest that in GaNSb, the interaction parameters, *β*_*N*_ and *β*_*NN*_, are 20% stronger than in GaNAs. Therefore, we multiply the N LCINS values by 1.44 to account for this difference. The blue solid line in the inset of Figure 9 presents the distribution of N states that we consider for GaN _0.012_Sb_0.988_ in our calculation.

Having the distribution of N states, we are able to calculate the Green’s function for GaN _0.012_Sb_0.988_ using Equations 19 and 20, self-consistently. Also, the density of CB states can be calculated using Equation 21. Figure 9 shows the DOS of GaN _0.012_Sb_0.988_ calculated by the SCGF method and including the distribution of localised states shown by the solid blue line in the inset of Figure 9. The gaps corresponding to isolated and pair N states are clearly observed in this plot. Also, at energies around 0.65 eV, the DOS has a small gap that is related to the higher cluster of N states.

We can also calculate the absorption spectrum using the SCGF model. The blue line with diamonds in Figure 8 shows the calculated absorption coefficient using this method. As expected, this method shows a better agreement with experiments than the result of the five-level BAC model (shown by red circles in this plot).

For the considered N distribution, this calculation suggests more gaps in the DOS of GaN _0.012_Sb_0.988_ compared to GaN _0.012_As_0.988_[2]. However, experimental data indicate that there are fewer features in the GaNSb absorption spectra than in the GaNAs ones. This could be due to inhomogeneities in the samples investigated experimentally, either due to fluctuations in the N composition in the experimental samples or because of intrinsic differences between the short-range N ordering in GaNSb and in InGaNAs samples.

Recent work by Mudd et al. [31] has shown that the composition dependence of the energy gap in GaN _*x*_Sb_1-*x*_ is well described using a three-level model including interactions between the host matrix band edge and the N isolated states and N-N pair states. The energy gap calculated using the LCINS model is also determined primarily by these interactions. The energy gap calculated here using the SCGF and LCINS method is consistent with experiment for the N composition *x*=1.2*%* which we consider and should closely follow the theoretical energy gap results presented in [31] as a function of N composition *x*.

## Conclusions

In this paper, we presented an analysis of the optical absorption spectra of dilute nitride alloys, calculated using the band structure model presented in our earlier work [2]. We have considered two different models to calculate the absorption spectra in InGaNAs and GaNSb alloys and compared our results with experimental measurements. We note however that there are some discrepancies between experimental data in similar samples that make quantitative comparison difficult.

Two models have been considered to calculate the absorption spectrum in these materials: a five-level BAC model and a LCINS-based model. The five-level BAC model included isolated and pair N states and their interactions with the host semiconductor valence and conduction bands. The results of this model for InGaNAs alloys give an overall good agreement with experiments, and predict accurate absorption edge for these alloys. However, the results of the five-level BAC model include several additional features not seen experimentally, supporting the need to consider a full distribution of N state energies in the electronic structure calculations.

We therefore extended our calculations to include the LCINS distribution using the SCGF approach presented in [2]. The calculated absorption spectra using this approach for InGaNAs provide very good agreement with experiments, supporting the validity of the LCINS approach to describe dilute nitride conduction band structure.

Our calculated absorption spectra for GaNSb alloys fit well with experiments at the absorption edge [31], and predict the correct band gap in these alloys. However, the absorption spectrum calculated in the BAC model contains features associated with individual transitions to lower and upper sub-bands in the model that are not seen in the measured absorption spectra. Taking the distribution of localised states into account reduces the impact of these features and gives results more similar to experimental absorption. But we still see some dips in our calculated spectra that are not seen in any experiment. We conclude that the distribution of N states in the GaNSb alloys studied are different from that for InGaNAs samples. We conclude that further work is required to address and resolve why more structure is found in the calculated absorption spectra compared to what is observed in the experimentally measured spectra.

## Competing interests

The authors declare that they have no competing interests.

## Authors’ contributions

SF proposed the SCGF approach to study the band structure of dilute nitride alloys. EOR suggested to apply this method to calculate the absorption spectrum. All calculations have been carried out by MS. All authors helped in drafting the manuscript. All authors read and approved the final manuscript.
